# Broadening the clinical spectrum for medical students towards primary care: a pre-post analysis of the effect of the implementation of a longitudinal clerkship in general practice

**DOI:** 10.1186/s12909-018-1152-z

**Published:** 2018-03-14

**Authors:** Roman Hari, Michael Harris, Peter Frey, Sven Streit

**Affiliations:** Institute of Primary Health Care Bern (BIHAM), University of Bern, Gesellschaftsstrasse 49, 3012 Bern, Switzerland

**Keywords:** Clinical teaching & learning. Primary care. Curriculum evaluation. Undergraduate education

## Abstract

**Background:**

Exposure to a broad spectrum of patient cases is a mainstay of undergraduate medical education. This study aimed to assess how many primary care-specific clinical pictures final-year medical students in traditional block rotations had encountered, and how this changed after a curricular change that included the implementation of a four-year longitudinal clerkship in primary care.

**Methods:**

Final-year students before, and after, implementation of the clerkship were asked which of the clinical pictures most relevant to primary care they had seen. We compared the overall proportions of clinical pictures seen by the two cohorts.

**Results:**

In the first cohort, 96 (66%) students responded, and 94 (65%) in the second. Before the curricular change, students had encountered a mean of 26.3 of the 34 primary care-specific clinical pictures (77.2%). After implementation of the longitudinal clerkship, this increased by 1.1 (4.2%, *P* = 0.038).

Among the eight clinical pictures seen the least by students in the first cohort, we found a significant increase in the proportion of students seeing polymalgia rheumatica, frozen shoulder, epicondylitis and Dupuytren’s contracture after the clerkship’s implementation.

**Conclusion:**

The undergraduate longitudinal clerkship in primary care broadened the spectrum of clinical pictures seen by medical students, to include more clinical pictures commonly seen in primary care.

**Electronic supplementary material:**

The online version of this article (10.1186/s12909-018-1152-z) contains supplementary material, which is available to authorized users.

## Background

Encountering a broad spectrum of patient cases in clinical work has long been a mainstay of undergraduate medical education [1]. The mixture of clinical conditions experienced in this setting is positively linked to positive learning experiences [2]. This is taken into account by the Swiss Catalogue of Learning Objectives for Undergraduate Medical Training (SCLO), a document that gives a list of the 1220 clinical pictures (the SCLO’s term for diagnostic entities) that need to be encountered and mastered by medical students during their training [3].

Whilst originally the training of doctors had taken place in the community, the main place for medical learning during the twentieth century became the hospital [4], leading to a shift in clinical pictures experienced by medical students, and underrepresentation in their curriculum of those clinical pictures more commonly encountered in primary care.

In longitudinal clerkships, students spend an extended time in a clinical setting, where they have continuity of patient contact and care, assessment and supervision, and clinical and cultural learning [5, 6]. These have been shown to have positive outcomes for students and patients, as well as supervising clinicians [7]. In recent decades, various undergraduate medical curricula have implemented longitudinal clerkships in general practice to broaden the educational spectrum for students [8]. This follows the 1988 Edinburgh Declaration of the World Federation for Medical Education, which advocated a substantial transfer of education from teaching hospitals to community settings [9]. Since then longitudinal clerkships have become recognised as representing credible and effective pedagogical alternatives to traditional block rotations in medical education [10]. In such clerkships, the majority of students have been found to be highly satisfied with their longitudinal placements [11], and students have done as well as, if not better than, their peers without such placements. In addition, in a longitudinal clerkship the continuity of contact with supervising doctors has been found to provide students with career mentorship and personal support, and students are more likely to report learning about chronic illness and communication skills, as well as receiving help in developing their clinical skills [12].

At the University of Bern in Switzerland, a curriculum reform has partially shifted the undergraduate clinical education setting from tertiary inpatients to community-based sites, and a mandatory four-year longitudinal clerkship in general practice was established in 2007 [13]. Within this scheme, students are assigned a general practitioner (GP) who acts as their mentor throughout the first four years of their studies. In group practices, students are assigned to one mentor only, however co-tutoring by other GPs in the same practice is common, especially to share interesting patient cases with the student or as substitute if the mentor GP is unavailable. If the assigned GP moves to a different practice, she or he is asked to supervise the student in their new practice. If the mentoring GP discontinues work, the practice is asked to continue supervision of the student. The model comprises eight half-days per year in the first three years, with a three-week primary care clerkship in the fourth year, all supervised by the same GP [13]. Before this curricular change there had been no mandatory training in general practice, and only 50% of students had chosen to take up an optional month-long clerkship in general practice in the final year of their training. The clerkship time in the hospital setting was reduced from 83 weeks to 49 weeks, including the removal of a three-week course in clinical dermatology. This overall reduction in clinical experience gave students time to write a Master’s thesis, as well as allowing more teaching time at the university campus, where lecture blocks in years 4–6 were expanded by 10 weeks to a total of 44 weeks.

The primary care longitudinal clerkships at the University of Bern differ considerably from some other longitudinal models. Whereas typical community placements in North America and Australia consist of at least 6 months spent continuously in a community setting [14, 15], our students only spend 27 days in primary care. This is mainly due to financial constraints. However, by distributing those days over 4 years, we have been able to establish sustained mentoring relationships between students and their assigned GPs.

While there is evidence that longitudinal clerkships in general practice have positive impacts on students’ career choices [8] and their likelihood of achieving various intended learning outcomes [16], we could find no previous evidence that directly assessed how the introduction of longitudinal clerkships has affected the spectrum of clinical cases seen by medical students. We therefore planned a pre-post analysis to assess how many primary care-specific clinical pictures listed in the SCLO the final-year medical students in traditional block rotations had encountered, and how that changed after the implementation of a four-year longitudinal clerkship in general practice.

## Methods

### Study design

We performed a pre-post analysis of data from cross-sectional surveys distributed to two groups of final year medical students, one cohort before and one after the implementation of a four-year longitudinal clerkship in general practice at the Medical Faculty of the University of Bern.

### Participants

We invited medical students in 2009 (before implementation of the longitudinal clerkship) and 2012 (after implementation) to participate in a paper survey that was distributed and collected after a lecture at the end of their final (sixth) year of medical school. A three-year gap between cohorts was chosen to allow comparison between the last group that had not undergone the longitudinal clerkship in primary care, and the first group that had completed it. We chose physical distribution of the questionnaires in order to maximise the response rate, although some students may have been absent for these lectures. Data collection was anonymous, therefore, according to Swiss Law, no ethics committee approval was required.

### Study variables

We developed a questionnaire for medical students in their final year to assess which of 34 clinical pictures from a pre-defined list they had seen in patient consultations at any time, and at any site, in their student years. The questionnaire also asked for the students’ gender, career intentions (‘GP, Specialist, Don’t know yet’), and their rating of the attractiveness of general practice and specialism as careers using a Likert-Scale (from 1, ‘Not attractive’, to 5, ‘Attractive’). As additional GP training was available to the medical students both before and after implementation of the longitudinal clerkship, we also asked whether they had taken that option up (‘Yes/No’). For an English translation of the questionnaire see Additional file 1.

We used a three-step approach to select the SCLO clinical pictures of most relevance to general practice. First, we selected the 414 clinical pictures that were already marked as ‘particularly relevant problems for General Practice and Outpatient Medicine’ in the SCLO list. After this, we invited five GPs who were experienced in teaching medical students to classify each of those 414 items as “Yes, relevant for general practice” or “No, not relevant for general practice”. Finally, in a face-to-face meeting, the five GPs shared their ratings. Clinical pictures rated by all five GPs as being relevant were kept in the list, those rated by all as irrelevant were removed from the list. Where there was no initial unanimity, there was discussion until a consensus was reached as to whether or not the items were relevant to general practice, and thus whether they should be kept on, or removed from, the list. Following this process, the list comprised 34 clinical pictures; each of these fitted into six of the SCLO’s thematic groups (Additional file 1).

### Statistical analysis

We compared the characteristics of the two cohorts (before and after implementation of the longitudinal clerkship) using chi-squared tests for categorical data, and *t* tests to compare continuous data. The proportions of clinical pictures seen by the cohorts were compared using a *t* test. We used logistic mixed-effects models to derive odds ratios (OR) with 95% confidence intervals (CI) to compare the proportions before and after implementation of the clerkship for crude data, and also for a multivariate model with data adjusted for gender, career intention, attractiveness of GP and specialism, and uptake of additional GP training. A mixed-effects model was used to account for the multiple assessments per student. To assess how the biggest primary care-specific gaps in achievement of the learning objectives in the former curriculum were affected by the implementation of the new curriculum, we performed a sub-analysis of the quartile of primary care-specific clinical pictures least seen by the 2009 cohort. For this we used chi-squared tests to make comparisons with the proportions seen by the 2012 cohort. We considered a *P* value of 0.05 or less to be statistically significant. We performed all analyses with STATA version 14.2 (Stata Corp, College Station, TX, USA).

## Results

In the 2009 cohort, 96 of 145 (66.2%) medical students completed the questionnaire. In the 2012 cohort, 94 out of 145 (65.8%) participated.

### Student characteristics

Table 1 compares the student characteristics of the two cohorts. Both samples replied similarly in terms of their career intentions and their rating of the attractiveness of general practice and specialism as careers. However, significantly more medical students in the pre-longitudinal clerkship cohort reported that they had taken up the option of additional GP training (49.0% in the 2009 cohort, 14.9% in the 2012 cohort, *P* < 0.001).

**Table 1:** Baseline Characteristics of the medical students before and after implementation of a longitudinal clerkship

Student characteristics	Before *n* = 96	After *n* = 94	*p*-value
Female, n (%)	68 (70.8)	54 (57.5)	0.054
Career intention, n (%)			0.41
GP	14 (14.6)	17 (18.5)	
Specialist	51 (53.1)	53 (57.6)	
Don’t know yet	31 (32.3)	22 (23.9)	
Career attractiveness rating^a^, score (SD)			
General Practice	3.4 (1.0)	3.4 (1.0)	0.57
Specialist	4.0 (0.7)	4.1 (0.8)	0.43
Additional GP training done, n (%)	47 (49.0)	14 (14.9)	< 0.001

^a^On a score from 1 (unattractive) to 5 (attractive)

### Student experience of primary care-specific clinical pictures before and after the implementation of a longitudinal clerkship

Students in the 2009 cohort had seen a mean of 26.3 of the 34 primary care-specific clinical pictures (77.2%) during their training. Table 2 shows the mean number of clinical pictures seen by students in each of six thematic groups. After the implementation of the longitudinal clerkships in general practice, students saw a mean of 27.4 primary care-specific clinical pictures (80.7%), an increase of 1.1 (4.2%) over the previous cohort (*P* = 0.038).

**Table 2:** Primary care-specific clinical pictures seen before and after implementation of a longitudinal clerkship

SCLO thematic group	Number of clinical pictures in group	Mean number of clinical pictures in group seen by medical students in 2009 cohort (%)	Mean number of clinical pictures in group seen by medical students in 2012 cohort (%)	*P*-Value
Internal medicine, musculo-skeletal	5	2.6 (51.0)	3.5 (70.6)	< 0.001
Internal medicine, non-musculo-skeletal	7	5.7 (80.8)	6.1 (87.1)	0.011
Surgery	9	6.5 (72.3)	6.8 (75.4)	0.22
Psychiatry	2	2.0 (97.9)	1.9 (94.0)	0.06
Dermatology	5	4.4 (88.5)	4.0 (79.6)	0.002
Otorhinolaryngology	6	5.2 (85.9)	5.2 (86.0)	0.98
Total	34	26.3 (77.2%)	27.4 (80.7)	0.038

Analysis of the clinical pictures in the six corresponding SCLO thematic groups shows a significant increase in the number of primary care-specific clinical pictures seen in musculo-skeletal disorders and internal medicine (non-musculo-skeletal) disorders. There was no significant difference in the number of clinical pictures seen by students in the surgical, psychiatric and otorhinolaryngological thematic groups. There was, however, a significant decrease the proportion of dermatological clinical pictures seen.

Using the multivariate model, the odds ratio for these clinical pictures being seen by students was 1.11 in favour of the 2012 cohort (95%CI 1.03 to 1.19).

### Changes in clinical pictures least seen by first cohort – Subgroup analysis

The eight clinical pictures seen least by the 2009 cohort were seen by a mean of 51.8% those medical students (Table 3), and this increased to 67.6% in the 2009 cohort (*P* = 0.004). The biggest increases were in polymalgia rheumatica (*P* < 0.001), frozen shoulder (*P* < 0.001), epicondylitis (*P* = 0.005) and Dupuytren’s contracture (*P* = 0.021) (Table 3).

**Table 3:** Proportion of clinical pictures least frequently seen, before and after implementation of a longitudinal clerkship

Clinical picture	Number of students seeing picture before implementation (%)	Number of students seeing picture after implementation (%)	Change in %	*P* value
Polymyalgia rheumatica	39 (40.6)	68 (72.3)	31.7%	< 0.001
Frozen shoulder	41 (42.7)	69 (73.4)	30.7%	< 0.001
Torticollis	48 (50.0)	51 (54.3)	4.3%	0.56
Bites	50 (52.1)	57 (60.6)	8.6%	0.24
Epicondylitis	53 (55.2)	70 (74.5)	19.3%	0.005
Dupuytren’s contracture	54 (56.3)	68 (72.3)	16.1%	0.02
Ganglion of wrist	56 (58.3)	60 (63.8)	5.5%	0.44
Burns	57 (59.4)	65 (69.1)	9.8%	0.16
Mean	49.8 (51.8)	63.5 (67.6)	15.7%	0.004

## Discussion

Medical students saw only three-quarters of the 34 primary care-specific clinical pictures in the former, secondary care-centred undergraduate medical curriculum in Bern. After implementation of a longitudinal clerkship in general practice, we found a significant increase in the overall number of primary care-specific clinical pictures students reported that they had seen. This increase was particularly high for the subset of clinical pictures that had been least encountered by the 2009 cohort. Analysis of thematic groups showed that the largest increase in clinical pictures seen was that of the primary care-specific musculo-skeletal diseases, which may reflect the high prevalence of musculo-skeletal disorders in primary care.

The overall increase of primary care specific clinical pictures seen by students was 1.1 after the implementation of the new curriculum. This increase is small, however our intervention was itself relatively small. Further research is required to assess whether the effect size will increase in line with the size of the intervention, e.g. the number of days spent in practice. The other curricular changes may also have affected the outcomes, but although total clerkship time decreased from 83 to 49 weeks, there was still an overall positive impact of the curricular change on primary care specific clinical pictures seen by students. The reduction in the proportion of dermatological clinical pictures seen by students may have been related to the removal of the clinical dermatology block from the undergraduate course, for which the new clerkship in primary care could not fully compensate. Usually there are few or no few patient contacts when writing a Master’s thesis, so we think it unlikely that this affected our primary endpoint. In the 2009 cohort, 49% of students reported that they had undergone additional (optional) GP training; this rate decreased significantly to 14.9% in the 2012 cohort, most likely due to shortening of the elective period in year 6 and better coverage of primary care in the overall curriculum. This difference may have reduced the effect of the curricular change. The study had a response rate that was sufficiently high to reduce, but not eliminate, the risk of selection bias. While asking medical students about their past experience might have introduced recall bias, this bias would be expected to have affected both samples. Although considerably more medical students in the first cohort had done additional GP training, both this and factors relating to career choice were adjusted for in the multivariate model. A weakness of our study is that, as our study only looked at primary care-related clinical pictures, we have no evidence as to whether the increase in the proportion of these clinical pictures seen was accompanied by a change in the proportion of secondary care-related clinical pictures seen.

Whereas some studies [10, 17], like ours, have reported on students’ own evaluations of their experience in longitudinal placements, other studies have used objective measures of skills. In a study using objective structured clinical examinations [18], students’ early clinical skill development was at least as good for those having community-based longitudinal placements as for those in traditional placements. A study reporting on licensing examination results found that a longitudinal integrated clerkship (LIC) was equivalent to, and on standardised patient examinations was slightly superior to, that of traditional peers [19]. In another study, students in a community-based LIC achieved significantly higher grades than those who undertook the traditional rotation programme [20].

Furthermore, in a comparison between students participating in a longitudinal clerkship and students in block rotations, there was no difference in clerkship grade, and students received similar clinical evaluations in both systems [21]. That study did, however, identify that the average written examination score was lower for students in the longitudinal clerkship. Other studies have examined the effect of longitudinal curricula on more generic outcomes. Longitudinal curriculum students performed at least as well as traditional students in tests of content knowledge and skills, they were more likely to receive feedback and mentoring from experienced faculty [22], and they have had higher satisfaction with faculty teaching and feedback [19].

Students in longitudinal primary care programmes have greater confidence in their behavioural, verbal and interpersonal skills than their peers in traditional programmes [17]. They also report more continuity-of-care experiences, more confidence in their quality improvement skills, and scored higher on measures of perceived patient centeredness [23]. There is also evidence that patients value the long-term engagement of medical students in their primary health-care teams [24].

## Conclusion

Our study provides evidence that longitudinal clerkships in general practice increases the number of GP-specific clinical pictures encountered by medical students compared to a traditional hospital-based curriculum, thereby broadening the spectrum of clinical pictures towards primary care in undergraduate medical education. Various aspects of the longitudinal clerkship design might have played an important role in this. Further research is required to ascertain which of these factors are the most effective in broadening the clinical spectrum of undergraduate medical education towards primary care.

## Additional file

Additional file 1: Training-spectrum survey. English translation of the original questionnaire developed for this study. (DOCX 21 kb)

## Abbreviations

BIHAM: Institute of Primary Health Care, University of Bern, Bern, Switzerland
CI: Confidence interval
GP: General practitioner
LIC: Longitudinal integrated clerkships
OR: Odds ratio
SCLO: Swiss Catalogue of Learning Objectives for Undergraduate Medical Training
