# Supplementary material for: Broadening the clinical spectrum for medical students towards primary care: a pre-post analysis of the effect of the implementation of a longitudinal clerkship in general practice
Source: BMC Med Educ. 2018 Mar 14;18:34. doi: 10.1186/s12909-018-1152-z (PMC5853096; doi:10.1186/s12909-018-1152-z)
Supplement: Supplementary file 1 — Training-spectrum survey. English translation of the original questionnaire developed for this study. (DOCX 21 kb) [file 12909_2018_1152_MOESM1_ESM.docx]

# Training-spectrum survey

Questionnaire for Human Medicine graduates, Bern

1. **Basic characteristics**
2. **Gender** 〇 male 〇 female
3. **What is your current career aspiration?** 〇 family doctor

〇 specialist doctor

〇 I do not know yet

1. **How attractive is the GP career to you?** not attractive ①②③④⑤ attractive
2. **How attractive is the specialist career to you?** not attractive ①②③④⑤ attractive
3. **During my studies, I completed further optional experience with a doctor in primary care** 〇 Yes 〇 No

**2. Training spectrum**

During my studies, I had **direct contact with patients** with the following conditions:

| **(Multiple answers possible)** | **At the GP practice** | **In the hospital** | **Never seen** |
| --- | --- | --- | --- |
| Fibromyalgia | 〇 | 〇 | 〇 |
| Periarthropathy of the shoulder | 〇 | 〇 | 〇 |
| Polymyalgia rheumatica | 〇 | 〇 | 〇 |
| Epicondylitis | 〇 | 〇 | 〇 |
| Torticollis | 〇 | 〇 | 〇 |
| Tension headaches | 〇 | 〇 | 〇 |
| Parkinson's disease | 〇 | 〇 | 〇 |
| Carpal tunnel syndrome (CTS) | 〇 | 〇 | 〇 |
| Thrombophlebitis | 〇 | 〇 | 〇 |
| First presentation of hypertension | 〇 | 〇 | 〇 |
| Chronic abdominal pain / IBD | 〇 | 〇 | 〇 |
| Appendicitis | 〇 | 〇 | 〇 |
| Pyelonephritis | 〇 | 〇 | 〇 |
| Depression | 〇 | 〇 | 〇 |
| Anxiety disorders | 〇 | 〇 | 〇 |
| Burns | 〇 | 〇 | 〇 |
| Boil, abscess | 〇 | 〇 | 〇 |
| Bite wound | 〇 | 〇 | 〇 |
| Ganglion | 〇 | 〇 | 〇 |
| Dupuytren's contractures | 〇 | 〇 | 〇 |
| Ingrown toenails | 〇 | 〇 | 〇 |
| Herpes simplex or H. zoster | 〇 | 〇 | 〇 |
| Seborrheic eczema | 〇 | 〇 | 〇 |
| Atopic eczema | 〇 | 〇 | 〇 |
| Actinic keratosis | 〇 | 〇 | 〇 |
| Fungal skin infections | 〇 | 〇 | 〇 |
| Lymphadenitis | 〇 | 〇 | 〇 |
| Evaluation of dizziness | 〇 | 〇 | 〇 |
| Otitis media | 〇 | 〇 | 〇 |
| Impacted earwax incl. ear irrigation | 〇 | 〇 | 〇 |
| Tonsillitis, by streptococci | 〇 | 〇 | 〇 |
| Pseudocroup (child) | 〇 | 〇 | 〇 |
| Pharyngitis | 〇 | 〇 | 〇 |
| Bronchitis | 〇 | 〇 | 〇 |
